# Allograft versus autograft for reconstruction after resection of primary bone tumors: a comparative study of long-term clinical outcomes and risk factors for failure of reconstruction

**DOI:** 10.1038/s41598-022-18772-x

**Published:** 2022-08-23

**Authors:** Taweechok Wisanuyotin, Permsak Paholpak, Winai Sirichativapee, Weerachai Kosuwon

**Affiliations:** Department of Orthopaedics, Faculty of Medicine, Khon Kaen University, Khon Kaen, 40002 Thailand

**Keywords:** Oncology, Risk factors

## Abstract

There have been no studies comparing the outcomes of nonvascularized autograft (NA) and allograft after resection of primary bone tumors. This study compares the clinical, functional outcomes of NA and allograft reconstruction and analyzes the risk factors for failure after these procedures. A retrospective study of patients with primary bone tumors of the extremities who underwent NA (n = 50) and allograft reconstruction (n = 47). The minimum follow up time was 24 months. The mean time to union for the NA and allograft group was 9.8 ± 2.9 months and 11.5 ± 2.8 months, respectively (*p* = 0.002). Reconstruction failure in the NA and allograft group was 19 (38%) and 26 (55.3%), respectively. Nonunion (30%) was the most common complication found in the NA group, while structural failure (29.8%) was the most common in the allograft group. There was no significant difference in functional outcome in terms of the mean Musculoskeletal Tumor Society score between the NA and allograft groups (23.5 ± 2.8 and 23.9 ± 2.1, respectively, p = 0.42). Age, sex, tumor location, graft length, method of reconstruction did not significantly influence failure of reconstruction. Chemotherapy was the only significant risk factor affecting outcomes (HR = 3.49, 95% CI = 1.59–7.63, *p* = 0.002). In the subgroup analysis, the use of chemotherapy affected graft-host nonunion (*p* < 0.001) and structural failure in both the NA and allograft groups (*p* = 0.02). Both NA and allograft reconstruction methods provide acceptable clinical and functional outcomes. Chemotherapy is a risk factor for failure of both reconstructions, particularly graft-host nonunion and structural failure.

## Introduction

Massive bone defect after resection of primary bone tumors remains a challenging problem. The treatment includes biological, endoprosthesis, and a combination of biological and endoprosthesis reconstructions. Each method has its advantages and disadvantages. The benefits of endoprosthesis reconstruction include its availability and early ambulation post-procedure. The disadvantages include complications that compound over time, such as aseptic loosening and bone loss^1^. Biological reconstruction has advantages in long-term use when host-bone graft incorporation is achieved, providing bone stock for future reconstruction and soft tissue attachment, thus improving joint kinematics and function^2,3^. The disadvantages include technical difficulty, prolonged operative time, and a high complication rate, including infection^4^, nonunion^5^, fracture, and joint degeneration^6^.

Biological reconstruction methods include vascularized and nonvascularized autograft (NA), allograft, recycled bone by freezing with liquid nitrogen, irradiation, autoclaving, and pasteurization^7^. NA and allograft reconstruction methods have long been used in biological reconstructions of the extremities. NA reconstruction is widely used for massive bone defects such as distal radius replacement, intercalary bone graft for diaphyseal bone defect of long bones, and resection arthrodesis procedure. The advantages of NA reconstruction are no risk of disease transmission or immune reaction and no special equipment required. The disadvantages of NA reconstruction include donor site pain, fracture, and the limited quantity of bone graft^8^. The use of allograft in bone tumor surgery is mostly from frozen allograft, which has an advantage over autograft in that there is no donor site morbidity or limit on quantity. The disadvantages of allograft include reducing osteogenicity and osteoconductivity due to processing and the risk of disease transmission^3,9^. For the best outcomes, bone allografts from organ donors should include a strict allograft process (namely, well-controlled harvesting, packaging, and storage in a bone bank) and optimal size-matching between the donor and host at the time of surgery^10,11^.

Although the literature reports high complication rates for biological reconstruction, favorable outcomes have also been reported^3,8,12–14^; however, no studies have been conducted comparing the NA and allograft reconstruction outcomes. We thus aimed to compare the clinical, functional outcomes and the failure rates of biological reconstruction of a massive bone defect after resection of primary bone tumors and reconstruction using either NA or allograft reconstruction. We also sought to determine the risk factors for the failure of these procedures.

## Patient and methods

A retrospective design study was performed after institutional review board approval was obtained (HE641241, Khon Kaen University, Thailand). Informed consent was obtained from all subjects or, if subjects are under 18, from a parent and/or legal guardian. All the protocol was performed in accordance with the relevant guidelines and regulations. Between January 1998 and December 2018, 68 patients underwent NA reconstruction, and 57 patients underwent allograft reconstruction after resection of primary bone tumors in the extremities.

The inclusion criteria were patients under 65 years of age, with a diagnosis of primary bone tumor in the extremities treated with limb-sparing surgery and reconstructed with structural bone graft (either NA or allograft). The exclusion criteria were patients who had metastasis at diagnosis, followed-up less than 2 years after surgery, or incomplete data.

Twenty-eight patients were excluded, 16 were followed up for less than two years, 6 died from tumor-related causes before the two-year follow-up, and 6 were lost to follow-up. Ninety-seven patients met the criteria, 50 for NA reconstruction and 47 for allograft reconstruction. The median age for the NA and allograft groups was 32.5 years (range, 11.9–61 years) and 24.8 (range, 7.9–53.1 years), respectively. The characteristics of the patients in both groups are presented in Table 1.

**Table 1:** Patient characteristics.

Variable		Nonvascularized autograft	Allograft
		(N = 50)	(N = 47)
Sex	Male	22 (44%)	22 (46.8%)
	Female	28 (56%)	25 (53.2%)
Age (years)	< 25	16 (32%)	24 (51.1%)
	≥ 25	34 (68%)	23 (48.9%)
Follow-up (months)	Median (range)	99.6 (24.3–216.6)	74 (24–219.6)
Diagnosis	GCT	24 (48%)	13 (27.7%)
	Osteosarcoma	21 (42%)	32 (68.1%)
	Chondrosarcoma	3 (6%)	0
	Malignant GCT	1 (2%)	1 (2.1%)
	Adamantinoma	0	1 (2.1%)
	Ewing’s sarcoma	1 (2%)	0
Location	Proximal humerus	0	4 (8.5%)
	Humeral shaft	2 (4%)	0
	Distal radius	3 (6%)	1 (2.1%)
	Femoral shaft	0	1 (2.1%)
	Distal femur	35 (70%)	22 (46.8%)
	Proximal tibia	10 (20%)	19 (40.4%)
Resection length (cm)	Mean	14.5 ± 3.2	16.2 ± 4.7
	< 15	22 (44%)	21 (44.7%)
	≥ 15	28 (56%)	26 (55.3%)
Chemotherapy	No	27 (54%)	17 (36.2%)
	Yes	23 (46%)	30 (63.8%)
Functional outcomes		23.5 ± 2.8	23.9 ± 2.1

In patients with osteosarcoma and Ewing sarcoma, the chemotherapy protocols (neoadjuvant chemotherapy) were administered before and after surgery, comprising combinations of methotrexate, doxorubicin, cisplatin, vincristine, ifosfamide, and etoposide depending on the year. Patients with giant cell tumor (GCT), malignant GCT, chondrosarcoma, or adamantinoma were treated by surgery only.

### Surgical procedures

**Figure 1:**
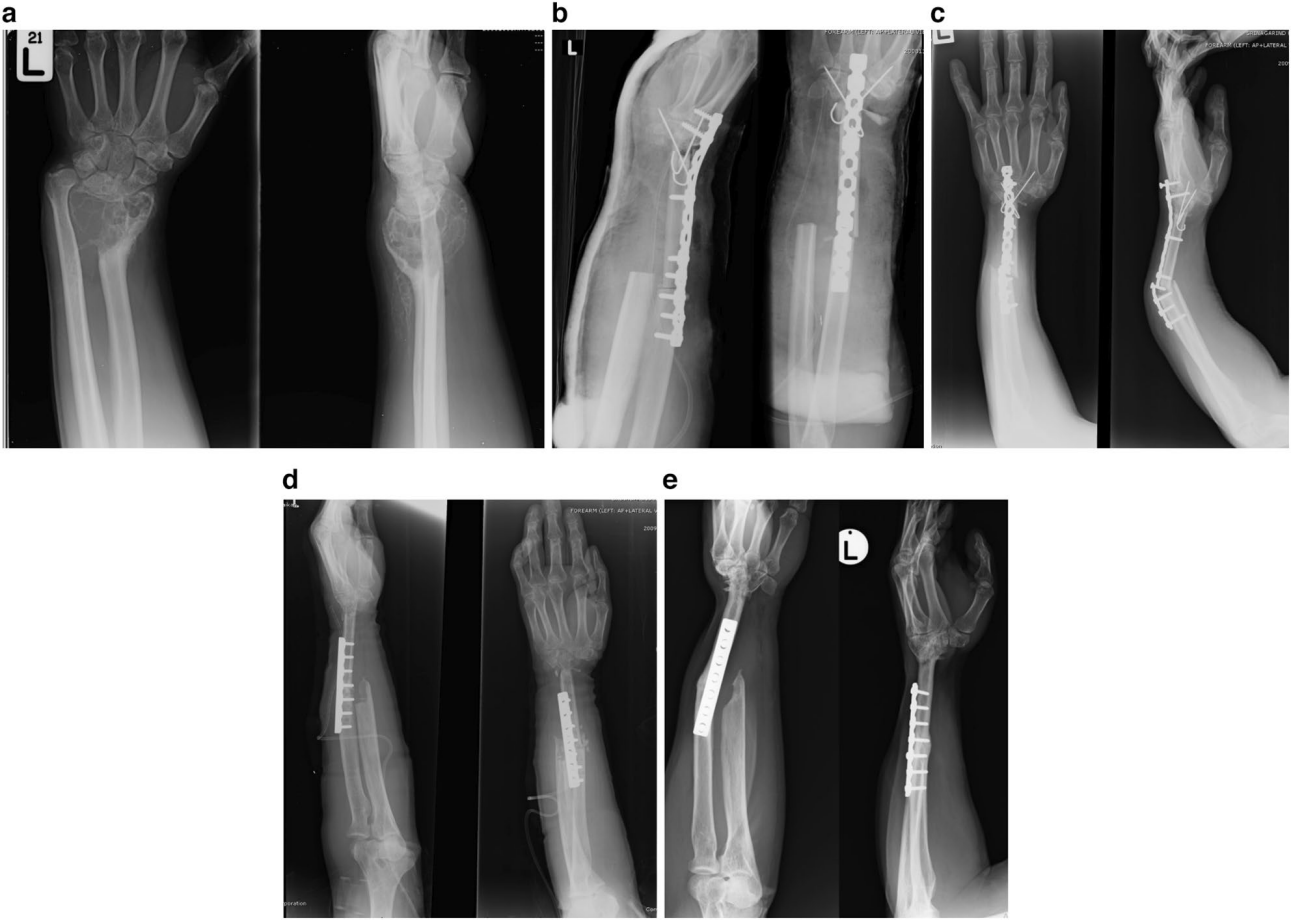
A 47-year-old male with giant cell tumor of the distal radius. (**a**) preoperative anteroposterior and lateral radiograph. (**b**) Postoperative radiograph after wide resection and reconstruction with nonvascularized fibula graft and fusion of the wrist joint. (**c**) Break of plate occurred 13 months postoperatively. (**d**) Revision surgery with plate and screws plus iliac bone graft, bone union was achieved in 9 months. (**e**) Three years after the revision surgery with acceptable functional outcome.

**Figure 2:**
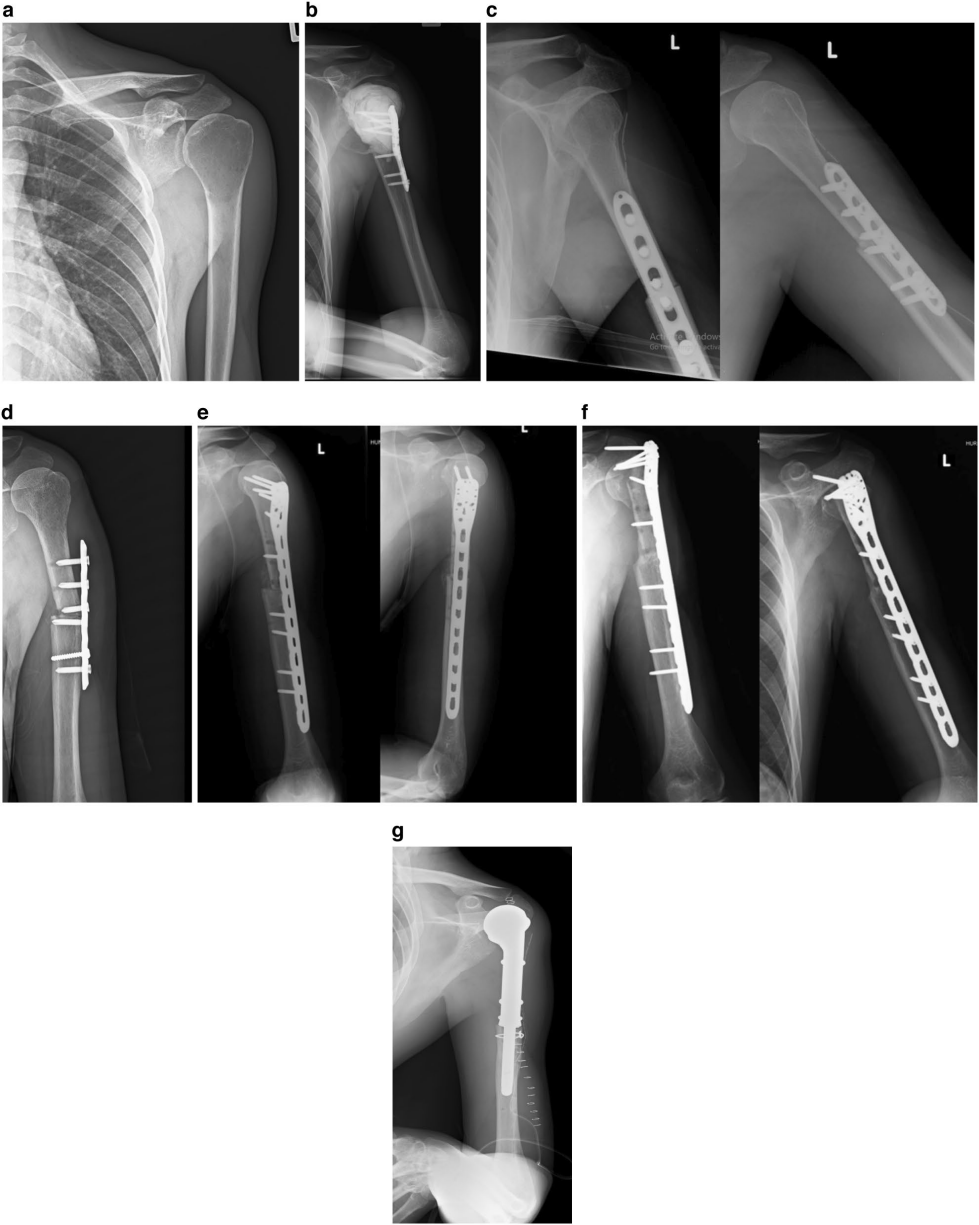
A 31-year-old male with giant cell tumor of the proximal humerus. (**a**) Pre-operative anteroposterior radiograph. (**b**) Six months after extended curettage with phenol and bone cement, tumor recurrence was detected. (**c**) Wide excision and allograft reconstruction was performed. (**d**) Three months later, failure of the implant was found. (**e**) Revision surgery with locking plate and screws was done. (**f**) Fifteen months after the revision procedure, fracture and lysis of the allograft occurred. (**g**) Finally, proximal humeral endoprosthesis was performed.

The choice of surgical procedures was discussed with the patients before surgery. In general, NA reconstruction was indicated for patients with the following; (1) Tumor at the distal radius, for which NA was harvested from the ipsilateral or contralateral proximal fibula to replace the bone defect as an osteoarticular bone graft (Fig. 1); (2) Tumor at the diaphysis of a long bone, for which the fibula shaft was harvested to fill the defect as an intercalary bone graft; and, (3) Tumor at the distal femur or the proximal tibia, for which the bone graft was harvested from the proximal tibia or the distal femur, respectively, and augmented with an ipsilateral fibula shaft to fill the defect as an intercalary bone graft (resection arthrodesis procedure).

Allograft reconstruction was indicated for treatment in patients with primary bone tumors at the diaphysis (intercalary allograft) or epiphysis-metaphysis (osteoarticular allograft) of the long bone (Fig. 2). The availability of allograft at the time of surgery, with size-matching between the host and graft, is a requirement for allograft reconstruction. Allografts were harvested under sterile conditions and stored frozen in our bone bank.

The extremities were immobilized in casts or braces for at least two months postoperatively or until bone union was achieved.

### Clinical and radiographic follow-up

Patients were followed up monthly for four months after surgery, then every two months for a year, every three months for two years, and every six months thereafter. At each follow-up, radiographs were analyzed until bone union was achieved. Bone union was defined as callus bridging at three or more cortices on the anteroposterior and lateral radiographs. Nonunion was defined as the presence of a radiolucent line at the host-graft junction after 12 months^5^.

At the final follow-up, the functional outcomes were assessed using the Musculoskeletal Tumor Society (MSTS) scoring system^15^, comprising six domains. The general factors are pain, function, and emotional acceptance. The specific factors for upper extremity include hand positioning, dexterity, and lifting ability. The specific factors for lower extremity include need for walking aids, walking, and gait. The score for each domain is 5 (range, 0–5). The total score, ranging from 0 to 30: the higher the score, the better the functional outcomes.

Postoperative complications were evaluated according to Henderson et al.^1^: Type 1 Soft-tissue failure, Type 2 Graft-host nonunion, Type 3 Structural failure (implant breakage or fracture), Type 4 Infection, Type 5 Tumor progression, and Type 6 Pediatric failure (physeal arrest or joint dysplasia).

### Statistical analysis

The continuous variables between the two groups were tested using the Mann–Whitney U test, while the categorical variables were tested with the Chi-square and/or Fisher's exact test.

Time to failure was defined as the interval between the date of the reconstructive surgery and the date of failure of the NA or allograft reconstruction. Graft survival was analyzed using the Kaplan–Meier survival analysis, and differences in survival between groups were compared using the log-rank test. The following variables were included in the study as independent risk factors: age^16,17^, sex^16,18^, tumor location^14,16,19^, graft length^17^, chemotherapy^5,16,20^, and method of reconstruction^17^. The independent risk factors for failure of biological reconstruction were determined using the Cox proportional hazards regression model. Bivariate and multivariable analyses as well as the crude and adjusted hazard ratios (HRs) with 95% confidence intervals (CIs) were calculated to present the effect of risk factors. Risk factors were considered statistically significant if *p* < 0.05. The statistical analyses were performed using SPSS version 23 (SPSS Inc., Chicago, IL, USA.).

## Results

Fifty patients with NA reconstruction were follow-up for a median time of 99.6 months (range, 24.3–216.6 months). The median time from the date of surgery to the date of failure was 37.9 months (range, 4.3–198.3 months).

Forty-seven patients with allograft reconstruction were followed up for a median time of 74 months (range, 24–219.6 months). The median time from the date of surgery to failure was 25.3 months (range, 3–103.1 months).

The mean length of bone graft was 14.5 ± 3.2 cm and 16.2 ± 4.7 cm in the NA and allograft group, respectively. The mean union time was 9.8 ± 2.9 and 11.5 ± 2.8 months in the NA and allograft group, respectively (*p* = 0.002). In the subgroup analysis of patients with benign and malignant bone tumors, the mean union time was 9.5 ± 2.6 months and 11.4 ± 3 months, respectively (*p* = 0.002).

### Failure of biological reconstruction

In the NA group, 38% (19 of 50) of patients developed a total of 26 complications. Nonunion was the most common complication in 30% (15 of 50), followed by structural failure in 16% (8 of 50), local recurrence in 4% (2 of 50), and infection in 2% (1 of 50) (Table 2). All patients with nonunion were successfully managed by cancellous autograft from the iliac crest. All patients with structural failure were managed by revising the implants (removal of an intramedullary nail and re-osteosynthesis with plating or revision with a locking plate). Deep infection was managed by multiple debridements and prolonged intravenous antibiotics. Tumor recurrence was treated by re-excision, which ultimately was unsuccessful so that amputation was required.

**Table 2:** Failure of biological reconstruction.

Variable	Nonvascularized autograft (N = 50)	Allograft (N = 47)
Total failures	26	37
Type 1 Soft-tissue failure	0	3
Type 2 Graft-host nonunion	15	12
Type 3 Structural failure	8	14
Type 4 Infection	1	5
Type 5 Tumor progression	2	2
Type 6 Pediatric failure	0	1

In the allograft group, 55.3% (26 of 47) of patients developed a total of 37 complications. Structural failure was found in 29.8% (14 of 47) followed by nonunion in 25.5% (12 of 47), infection in 10.6% (5 of 47), soft tissue failure in 6.4% (3 of 47), local recurrence in 4.3% (2 of 47), and physeal arrest in 2.1% (1 of 47) (Table 2). All patients who had structural failure needed revision of the implants and all patients with nonunion were treated with cancellous autograft from the iliac crest. In patients with an infection, the successful treatment consisted of multiple debridements with prolonged intravenous antibiotics. In two patients with instability, one was treated with arthrodesis and the other with an endoprosthesis reconstruction.

At the final follow-up, three patients in the NA group (two for tumor recurrence and one for infection) and four in the allograft group (two for structural failure, one for tumor recurrence and one for nonunion) required amputation. In addition, five patients in the allograft group had been converted to an endoprosthesis reconstruction (one for instability and four for intraarticular fracture of the allograft). The overall retention rate of the limb in the NA and allograft group was 94% and 80.9%, respectively.

### Risk factors for failures of biological reconstruction

The risk factors for failure of biological reconstruction were analyzed by bivariate and multivariable analyses (Table 3). In the bivariate analysis, method of reconstruction (*p* = 0.005) and the use of chemotherapy (*p* < 0.001) were associated with the failure of biological reconstruction. However, in the multivariable analysis, chemotherapy was the only factor significantly associated with failure of biological reconstruction (*p* = 0.002, HR = 3.49, 95% CI = 1.59–7.63).

**Table 3:** Prognostic factors for failure of biological reconstruction.

Variable	Crude			Adjusted*		
	*p* value	HR	95% CI	*p* value	HR	95% CI
**Age**						
< 25		1	(reference)	(reference)		
≥ 25	0.12	0.62	0.34–1.12	0.52	1.25	0.64–2.42
**Sex**						
Male		1	(reference)	(reference)		
Female	0.19	0.68	0.38–1.22	0.41	0.76	0.4–1.44
**Tumor location**						
Upper extremity		1	(reference)	(reference)		
Lower extremity	0.46	1.56	0.48–5.05	0.35	1.79	0.52–6.19
**Graft length**						
< 15 cm		1	(reference)	(reference)		
≥ 15 cm	0.35	0.76	0.42–1.36	0.29	0.7	0.36–1.37
**Method of reconstruction**						
Autograft		1	(reference)	(reference)		
Allograft	0.005	2.37	1.3–4.33	0.09	1.71	0.91–3.2
**Chemotherapy**						
No		1	(reference)	(reference)		
Yes	< 0.001	3.95	1.99–7.82	0.002	3.49	1.59–7.63

*HR*  Hazard ratio, *CI*  confidence interval. *Adjusted for age, sex, tumor location, graft length, method of reconstruction, and the use of chemotherapy.

The association between the use of chemotherapy and the failure of reconstruction was analyzed in subgroup analysis. There was a statistically significant difference between patients who received chemotherapy and those who did not receive chemotherapy in graft-host nonunion (41.5% and 11.4%, *p* < 0.001) and structural failure (32.1% and 11.4%, *p* = 0.02) (Table 4).

**Table 4:** Subgroup analysis of patients according to the use of chemotherapy.

	No chemotherapy (N = 44)	Chemotherapy (N = 53)	*p* value
Time to union (months)	9.4 ± 2.7	11.6 ± 2.8	< 0.001
**Failure of reconstruction **			
Total failure (n)	12 (27.3%)	33 (62.3%)	< 0.001
Type 1 Soft-tissue failure	1	2	0.67
Type 2 Graft-host nonunion	5	22	< 0.001
Type 3 Structural failure	5	17	0.02
Type 4 Infection	3	3	0.81
Type 5 Tumor progression	1	3	0.41
Type 6 Pediatric failure	0	1	N/A

*N/A*  Not applicable.

### Survival analysis

**Figure 3:**
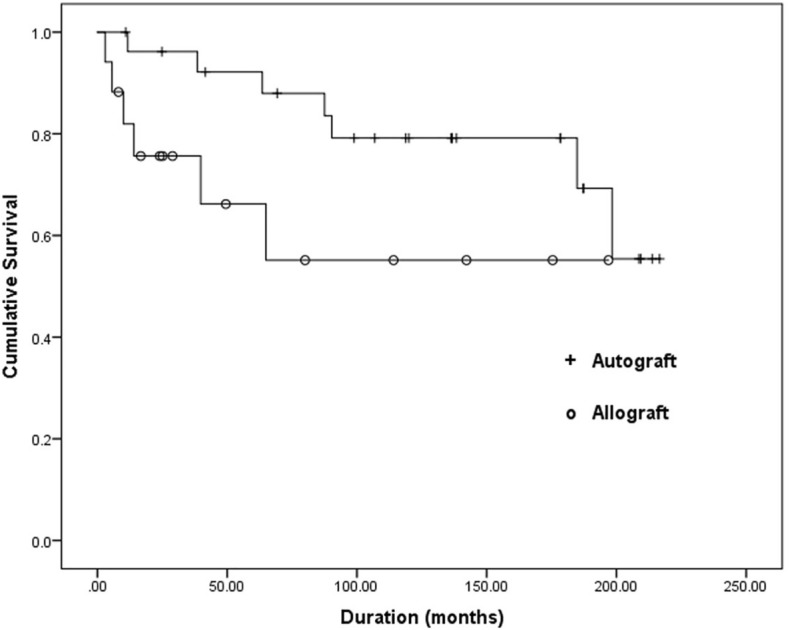
Kaplan–Meier survival curve of the bone graft in the subgroup of patients who did not receive chemotherapy. There was no statistically significant difference between the nonvascularized autograft and allograft groups (*p* = 0.06).

**Figure 4:**
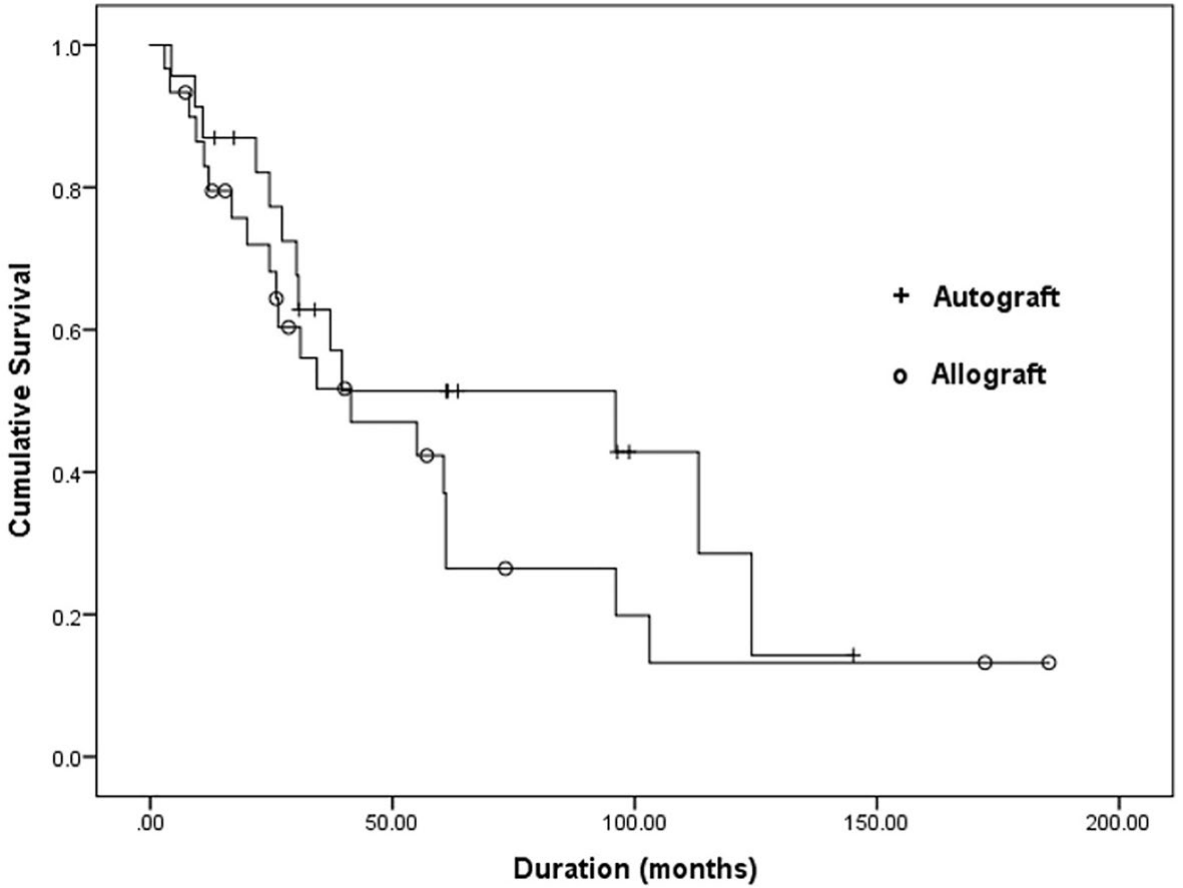
Kaplan–Meier survival curve of the bone graft in the subgroup of patients who received chemotherapy. There was no statistically significant difference between the nonvascularized autograft and allograft groups (*p* = 0.27).

Based on analysis of the risk factors for failure of biological reconstruction, chemotherapy was considered the only factor that influenced graft survival, so the NA and allograft groups then underwent a subgroup analysis for the use of chemotherapy. The survival analyses using the Kaplan–Meier curve for both groups are presented in Figs. 3 and 4.

For patients who did not receive chemotherapy, the 5- and 10-year overall survival rate of the graft in the NA group was 88% and 79%, respectively. While in the allograft group, the 5- and 10-year overall survival rate of the graft was 55% and 55%, respectively (*p* = 0.06).

For patients who received chemotherapy, the 5- and 10-year overall survival rate of the graft in the NA group was 51% and 13%, respectively. As for the allograft group, the 5- and 10-year overall survival rate of the graft was 27% and 13%, respectively (*p* = 0.27).

The overall mean union time in both groups who received chemotherapy was 11.6 ± 2.8 months and 9.4 ± 2.7 months for those who did not receive chemotherapy (*p* < 0.001). When the subgroup analysis was performed in patients who did not receive chemotherapy, the mean union time in the NA and allograft groups was 8.4 ± 2.1 months and 10.9 ± 2.9 months, respectively (*p* = 0.002). In patients who received chemotherapy, the mean union time in the NA and allograft groups was 11.4 ± 2.9 months and 11.9 ± 2.8 months, respectively (*p* = 0.53).

### Functional outcomes

The mean functional outcomes at the final follow-up in the NA and allograft groups was 23.5 ± 2.8 and 23.9 ± 2.1, respectively. There was no statistically significant difference between treatment with NA or allograft reconstruction methods (*p* = 0.42).

## Discussion

Reconstruction of massive bone defects after resection of bone tumors remains a challenge for orthopedic oncologic surgeons. To select the appropriate technique, we must first understand the long-term clinical outcomes and the complication rate of the chosen reconstruction method. Therefore, we analyzed NA and allograft reconstruction outcomes and identified the risk factors that influenced the failure of both reconstruction methods.

In bone tumor surgery, the structural bone graft, which provides structural support and mechanical strength, is required to replace the massive resected bone tumor. Autogenous bone graft is the gold standard among all bone graft materials due to its histocompatibility, osteogenicity, osteoinductivity, and osteoconductivity^21^. In addition, autogenous bone graft has several advantages, including no risk of disease transmission, short union time, and low cost; notwithstanding, donor site morbidity and limited graft material are disadvantages^8^. Allograft is an alternative option for treating the massive bone defect as it provides adequate bone graft material without donor site morbidity; however, its osteoinductive and osteogenicity properties are limited^9^.

Schuh et al.^22^ compared the results of vascularized and nonvascularized fibula graft reconstruction after resection of a bone tumor. They reported no difference between groups in achieving bone union except that the vascularized autograft required more revision surgery.

Aithal et al.^12^ reported 20 cases of giant cell tumors of the distal radius treated by tumor resection and reconstruction with nonvascularized fibular osteoarticular autograft. The mean union time was 5.2 months (range, 4–6.5 months). Nonunion was found in 3 cases. These results were better than our series which had a mean union time of 9.8 ± 2.9 months and a 30% postoperative complication rate of nonunion, and a 16% rate of structural failure. This may be related to the chemotherapy rate, which was not stated. Krieg et al.^8^ analyzed 31 patients treated with a nonvascularized fibular graft after resection of primary bone tumors. The union rate of 89% was achieved in a median time of 24 weeks (range, 7–61 weeks). The fracture of the graft was found in 19%. The chemotherapy or radiotherapy had a significant adverse effect on union of the graft. In resection arthrodesis of the knee, which the NA was used to fill the defected in the distal femur or the proximal tibia, Enneking et al.^23^ reported 20 patients with primary bone tumors around the knee. The structural failure occurred in 25%, nonunion in 20%, and 5% local recurrence. Salai et al.^24^ reported nine patients who underwent resection arthrodesis of the knee after resection of bone tumors. The mean union time was 19 weeks. The complications were one patient each for infection and local recurrence.

The allograft reconstruction in our series had a mean union time of 11.5 ± 2.8 months with a rate of post-operative complications of a structural failure of 29.8% and a nonunion of 25.5%. Mankin et al.^11^ reported a large series of 718 patients who underwent allograft reconstruction with at least 2 years of follow-up. Fracture was the most common complication (19%), followed by nonunion (17%), infection (11%), and joint instability (6%). The results from Mankin et al. were better than our series possibly because of better allograft quality or lower chemotherapy rate. Ogilvie et al.^6^ retrospectively reviewed 20 patients who underwent primary osteoarticular allograft reconstruction after resection of bone tumors with a minimum follow-up of 10 years. Seventy percent of patients had postoperative complications: fracture (45%), progressive arthritis (25%), nonunion (20%), and infection (10%). Sixty percent of allografts were removed at an average of 5.2 years. Bus et al.^13^ retrospectively reviewed 87 patients from a multicenter study of intercalary allograft reconstructions following resection of primary bone tumors. Postoperative complications were nonunion (40%), fracture (29%), and infection (14%). Reconstruction site, age, allograft length, nail-only fixation, and non-bridging osteosynthesis were associated with risk factors for complications. Adjuvant chemotherapy and irradiation had no effects on complication rates.

The current study revealed a high complication rate of 38% in the NA group and 55.3% in the allograft group. However, it should be noted that the number of patients who received chemotherapy in the allograft group was greater than those patients in the NA group. Moreover, univariate and multivariable analyses revealed that either NA or allograft reconstruction did not affect postoperative complications. Therefore, only chemotherapy was considered a significant variable influencing outcomes, explaining why the allograft group had higher complication rates than the NA group. Thus, we conducted a further subgroup analysis of patients based on their use of chemotherapy. We found that chemotherapy significantly affected the complications in graft-host nonunion and structural failure. However, the use of chemotherapy remains controversial in its effect on complications following the biological reconstruction^2,5,8,13,20,22^. Our results confirm previous reports indicating that chemotherapy results in a higher rate of nonunion^5,20^.The effect of chemotherapy on bone healing was studied in animal models. Eisenschenk et al.^25^ compared the effect of chemotherapy on bone healing of vascularized rib and fibula grafts in dogs. They found 100% union rate of bone graft in those that did not receive chemotherapy compared to 30% of vascularized rib and 80% of fibula grafts among those that received chemotherapy. The effect of chemotherapy on segmental bone healing was studied in rabbits^26^: the results showed that chemotherapy affected both the quantity and quality of bone enhanced by rhBMP-2.

Hazan et al.^20^ studied the effect of chemotherapy on complications after osteoarticular allograft reconstruction. The rate of nonunion was higher in patients who received chemotherapy (32%) than in those who did not (12%). Hornicek et al.^5^ reported a 27% nonunion rate for an allograft-host junction in patients who received chemotherapy compared with 11% for those who did not.

Donati et al. ^27^ conducted a review of 112 allograft reconstructions. The most common postoperative complications were delayed union (49%) and fracture (27%). In addition, they found that chemotherapy increased the likelihood of delayed union. Bus et al.^13^ retrospectively reviewed 87 patients with intercalary allograft reconstruction. The most common complications were nonunion (40%), fracture (29%), and infection (14%). Adjuvant chemotherapy and irradiation had no effects on complication rates. The risk factors for complications were site, age, allograft length, nail-only fixation, and non-bridging osteosynthesis.

In our study, the NA group had a shorter union time than the allograft group. However, when a subgroup analysis according to chemotherapy was performed, a longer union time was found in patients who received chemotherapy over against those who did not. There was no significant difference in union time between the NA and allograft group in the subgroup of patients who received chemotherapy. In the subgroup of patients who did not receive chemotherapy, the NA group had a shorter union time than the allograft group. Our findings confirm previous studies in which NA had a shorter union time than allograft and that chemotherapy delayed bone union^2,8,12,24,27,28^.

The literature indicates that the survival rate of the allograft varies widely. Bus et al.^14^ retrospectively reviewed 38 patients with primary bone tumor and reconstruction with osteoarticular allograft. The 5- and 10- year of the allograft survival was 52% and 41%, respectively. Ogilvie et al.^6^ retrospectively reviewed 20 patients with primary bone sarcoma who were followed up at least 10 years. The 5- and 10- year osteoarticular allograft survival was 55% and 34%, respectively. Muscolo et al.^29^ studied 76 patients with a primary bone tumor. The survival rate of the distal femoral osteoarticular allograft was 78% at both 5 and 10 years. As for the survival rate of the nonvascularized autograft, Schuh et al.^22^ reported a reconstruction after resection of primary sarcoma. The revision-free survival at 5 years was 55.6%. The survival rates in our study were lower than in previous studies because the definition of failure of reconstruction that we used, based on Henderson et al.^1^, while in other studies^6,13,14,29^, it was defined as removal of the graft, necessitating a revision procedure or an amputation. In our study, the survival rate of the bone graft was categorized according to the use of chemotherapy. We found that in patients who received chemotherapy, the graft survival of both the NA and allograft groups was significantly worse than in those patients who did not receive chemotherapy. This survival rate confirms that the failure of the reconstruction is adversely affected by chemotherapy.

Our study has several limitations. First, it is retrospective, so direct comparisons are limited. Second, due to the rarity of the disease, the sample size was small and heterogeneous. A future study should be performed to demonstrate the effect of chemotherapy on the failure of biological reconstruction that includes more patients and/or is multicenter. Third, chemotherapy regimens have changed over the years as the science develops and can vary among diseases, thus affecting reconstruction outcomes. Fourth, the long-term nature of the study may have been confounded by the evolution of surgical techniques over the years.

## Conclusions

After resection of primary bone tumors, NA and allograft reconstruction provide satisfactory functional outcomes despite several anticipated complications. Either method can be used without significant differences in graft survival and complications. Chemotherapy influences the failure of biological reconstruction, particularly graft-host nonunion and structural failure. Thus, in cases of malignant bone tumors undergoing chemotherapy, the choice of biological reconstruction with NA and allograft reconstruction should be used with caution.

## Data Availability

The data that support the findings of this study are available on reasonable request from the corresponding author.

## References

[1] Henderson ER (2014). Classification of failure of limb salvage after reconstructive surgery for bone tumours: A modified system Including biological and expandable reconstructions. The Bone Joint J..

[2] Ortiz-Cruz E, Gebhardt MC, Jennings LC, Springfield DS, Mankin HJ (1997). The results of transplantation of intercalary allografts after resection of tumors. A long-term follow-up study. The J. Bone Joint Surg..

[3] Temple HT (2019). Allograft reconstruction of the knee-methods and outcomes. J. Knee Surg..

[4] Mankin HJ, Hornicek FJ, Raskin KA (2005). Infection in massive bone allografts. Clin. Orthop. Relat. Res..

[5] Hornicek FJ (2001). Factors affecting nonunion of the allograft-host junction. Clin. Orthop. Relat. Res..

[6] Ogilvie CM, Crawford EA, Hosalkar HS, King JJ, Lackman RD (2009). Long-term results for limb salvage with osteoarticular allograft reconstruction. Clin. Orthop. Relat. Res..

[7] Yamamoto N, Hayashi K, Tsuchiya H (2019). Progress in biological reconstruction and enhanced bone revitalization for bone defects. J. Orthop. Sci.: Off. J. Japanese Orthop. Assoc..

[8] Krieg AH, Hefti F (2007). Reconstruction with non-vascularised fibular grafts after resection of bone tumours. The J. Bone Joint Surg..

[9] Delloye C, Cornu O, Druez V, Barbier O (2007). Bone allografts: What they can offer and what they cannot. The J. Bone Joint Surg..

[10] Fu SH (2017). Quality control processes in allografting: A twenty-year retrospective review of a hospital-based bone bank in Taiwan. PLoS ONE.

[11] Mankin HJ, Gebhardt MC, Jennings LC, Springfield DS, Tomford WW (1996). Long-term results of allograft replacement in the management of bone tumors. Clin. Orthop. Relat. Res..

[12] Aithal VK, Bhaskaranand K (2003). Reconstruction of the distal radius by fibula following excision of giant cell tumor. Int. Orthop..

[13] Bus MP (2014). Intercalary allograft reconstructions following resection of primary bone tumors: a nationwide multicenter study. The J. Bone Joint Surg..

[14] Bus MP, van de Sande MA, Taminiau AH, Dijkstra PD (2017). Is there still a role for osteoarticular allograft reconstruction in musculoskeletal tumour surgery? A long-term follow-up study of 38 patients and systematic review of the literature. The Bone Joint J..

[15] Enneking WF, Dunham W, Gebhardt MC, Malawar M, Pritchard DJ (1993). A system for the functional evaluation of reconstructive procedures after surgical treatment of tumors of the musculoskeletal system. Clin. Orthop. Relat. Res..

[16] Ippolito JA (2019). Complications following allograft reconstruction for primary bone tumors: Considerations for management. J. Orthop..

[17] Frisoni T, Cevolani L, Giorgini A, Dozza B, Donati DM (2012). Factors affecting outcome of massive intercalary bone allografts in the treatment of tumours of the femur. The J. Bone Joint Surg..

[18] Aponte-Tinao LA, Ayerza MA, Muscolo DL, Farfalli GL (2016). What are the risk factors and management options for infection after reconstruction with massive bone allografts?. Clin. Orthop. Relat. Res..

[19] Aponte-Tinao LA, Ayerza MA, Albergo JI, Farfalli GL (2020). Do Massive allograft reconstructions for tumors of the femur and Tibia survive 10 or more years after implantation?. Clin. Orthop. Relat. Res..

[20] Hazan EJ, Hornicek FJ, Tomford W, Gebhardt MC, Mankin HJ (2001). The effect of adjuvant chemotherapy on osteoarticular allografts. Clin. Orthop. Relat. Res..

[21] Myeroff C, Archdeacon M (2011). Autogenous bone graft: Donor sites and techniques. The J. Bone Joint Surg..

[22] Schuh R (2014). Vascularised or non-vascularised autologous fibular grafting for the reconstruction of a diaphyseal bone defect after resection of a musculoskeletal tumour. The Bone Joint J..

[23] Enneking WF, Shirley PD (1977). Resection-arthrodesis for malignant and potentially malignant lesions about the knee using an intramedullary rod and local bone grafts. The J. Bone Joint Surg..

[24] Salai M, Nerubay J, Caspi I, Horoszowski H (1997). Resection arthrodesis of the knee in the treatment of tumours–a long-term follow-up. Int. Orthop..

[25] Eisenschenk A (2007). Does chemotherapy impair the bone healing and biomechanical stability of vascularized rib and fibula grafts?. J. Reconstr. Microsurg..

[26] Morcuende JA (2004). Effect of chemotherapy on segmental bone healing enhanced by rhBMP-2. Iowa Orthop. J..

[27] Donati D (2000). Massive bone allograft reconstruction in high-grade osteosarcoma. Clin. Orthop. Relat. Res..

[28] Liu Q (2020). Intercalary allograft to reconstruct large-segment diaphysis defects after resection of lower extremity malignant bone tumor. Cancer Manag. Res..

[29] Muscolo DL, Ayerza MA, Aponte-Tinao LA, Ranalletta M (2005). Use of distal femoral osteoarticular allografts in limb salvage surgery. The J. Bone Joint Surg..

